# Male Copulatory Structures in Reproductively Functional Female Live‐Bearing Fish *Pseudopoecilia fria*

**DOI:** 10.1002/ece3.73118

**Published:** 2026-02-17

**Authors:** Justin Yeager, Leonardo Avila, Callen Inman, Marissa Cartee, Micaela Pozo, Dillan Burbano

**Affiliations:** ^1^ Grupo de Investigación en Biodiversidad, Medio Ambiente y Salud (BIOMAS), Facultad de Ingenierías y Ciencias Aplicadas Universidad de Las Américas Quito Ecuador; ^2^ Dirección de Investigación y Vinculación, Escuela de Biotecnología, Facultad de Ingenierías y Ciencias Aplicada Universidad de Las Américas Quito Ecuador; ^3^ Department of Integrative Biology University of Texas at Austin Austin Texas USA; ^4^ Department of Evolution, Ecology and Organismal Biology University of California at Riverside Riverside California USA

## Abstract

The presence of sexual characteristics typical of one sex in the opposite sex is more common than has been previously recognized. When changes in the environment or the genome alter sex‐specific regulatory processes, individuals may develop novel reproductive phenotypes. We recently discovered a population of the live‐bearing fish *Pseudopoecilia fria* (Cyprinodontiformes: Poeciliidae) in which females were found to possess gonopodia, reproductive organs normally exclusive to male fish that are used in the transfer of sperm in mating. While we are not yet able to identify the specific mechanisms underlying this phenotype, we tentatively attribute it to female masculinization. We document that this process has occurred in a significant proportion of the females sampled in the population. Geometric morphometric analyses showed that females were potentially divided into two discrete phenotypes, though with considerable intra‐group variance. Additionally, all females with gonopodia were gravid, and one individual gave birth to live offspring. Therefore, these alternate female morphs appear reproductively functional. We discuss several potential explanations for this phenomenon, including exposure to masculinizing water‐borne pollutants and the remote possibility of a fixed polymorphism. Additionally, we suggest a number of lines of research which could be motivated by this discovery.

## Graphical abstract

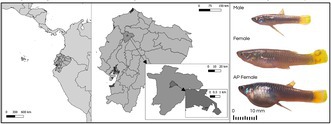

## 1 Introduction

In many organisms with separate sexes, mature males and females are clearly discriminable based on both primary and secondary sexual characteristics. Sexual differentiation can be influenced by multiple sources, and determination is a delicate process subject to endocrine/hormonal regulation (Yanong et al. 2006), variation in autosomal gene expression, novel mutations, and environmental perturbation (Massa et al. 2023). Indeed, developmental abnormality in the expression of primary sexual characteristics may lead to cross‐sexual transfer (West‐Eberhard 2003; Anderson and Falk 2023), in which traits expressed exclusively in one sex in ancestors appear in the opposite sex of descendants. The idea of cross‐sexual transfer, first noted by Darwin (1871) and expanded by West‐Eberhard (2003), suggests that sex‐specific regulation underlies the vast majority of sex differences and that novel phenotypes with important evolutionary implications may be acquired through relatively minor alterations to the genome or even (at least initially) via environmental changes.

Although most vertebrates are gonochoristic, where each individual develops sex organs for producing either male or female gametes, hermaphroditism, in which an individual has reproductive organs for producing male or female gametes, has evolved in several fish lineages (Erisman et al. 2013; Pla et al. 2022). Hermaphroditism may be bidirectional, induced by specific social or ecological cues, or occurring as a fixed series (St. Mary 1994, 1997; Waples et al. 2018; Benvenuto et al. 2020; Casas and Saborido‐Rey 2021). The proximate underlying causes of cross‐sexual transfer are diverse but can include pathologies induced by parasites on their hosts that can induce feminization or masculinization (Vance 1997), stress‐related mutations (Walbot et al. 1987), and very high estrogen levels that induce female‐like development in sexual mimics (Moore 1991).

Cross‐sexual transfer is often caused by changes in environmental cues that in turn alter hormonal exposure. Masculinization, the expression of male‐typical morphological structures in females, has been found in a number of fish species and can be induced using exogenous androgens (e.g., synthetic steroid 17α‐Methyltestosterone, Pandian and Sheela 1995; Beardmore et al. 2001), or blocking aromatase activity (Tran et al. 2022). Induced masculinization could have an effect in population‐wide sex ratios, even causing entire population loss (Senior et al. 2016). Similarly, feminization, the expression of female characteristics in males, can be achieved by exogenous estrogen (Piferrer 2001). The induction of masculinization and feminization with hormonal treatments has long been practiced to manage sex ratios in aquaculture (Pandian and Sheela 1995), but masculinization and feminization can also result from incidental anthropogenic influence in the form of domestic and industrial effluent. Freshwater ecosystems are frequently affected by human influenced modification of the environment, and wastewater effluent from urban environments or agriculture can alter assemblage of fish populations (Araújo and Tejerina‐Garro 2009; Mondal and Bhat 2020). Endocrine disrupting chemicals (EDC) can affect metabolic pathways in the endocrine system, producing changes in gonadal formation, embryo development and sex differentiation (Zheng et al. 2015) and are present in waterways from agriculture, and could be involved in the prevalence of alternate sex phenotypes in fish (testicular oocytes in smallmouth bass, *Micropterus dolomieu* , Kadlec et al. 2017).

Likewise, many pollutants can have androgenic effects on fish, causing developing females to undergo masculinization. In the freshwater poeciliid fish, *Cnesterodon decemmaculatus* , over 80% of females sampled downstream of an urban‐industrial area had developed male intromittent organs (gonopodia) and exhibited male mating behavior (Vidal et al. 2018). Masculinization and feminization can result in negative demographic consequences for populations. Documenting novel masculinized and feminized phenotypes in natural populations will help us to understand the mechanisms underlying their emergence, whether anthropogenic or naturally occurring, as well as broader impacts on fitness and population viability.

Poeciliids (Order: Cyprinodontiformes) are freshwater and euryhaline teleost fishes which often display conspicuous sexual traits: males of some species have elaborate ornaments and courtship displays, and all males have modified anal fins (gonopodia) or laterally compressed bodies (Furness et al. 2019). Numerous other cases of cross‐sexual transfer in the form of masculinization have been reported or experimentally induced in poeciliids, attributable to exogenous androgens (Angus et al. 2001; Yanong et al. 2006; Chakraborty et al. 2012; Vidal et al. 2018; Tran et al. 2022). Cases of masculinization in poeciliids typically report the development of male copulatory (gonopodia) and testicular structures, and, intriguingly, male mating behavior in females (Vidal et al. 2018; Tran et al. 2022). While masculinized female poeciliids typically do not possess functional male gonads, protogynous sex change has been recorded in domestic strains of *Xiphophorus helleri* (Lodi 1980).

**FIGURE 1:**
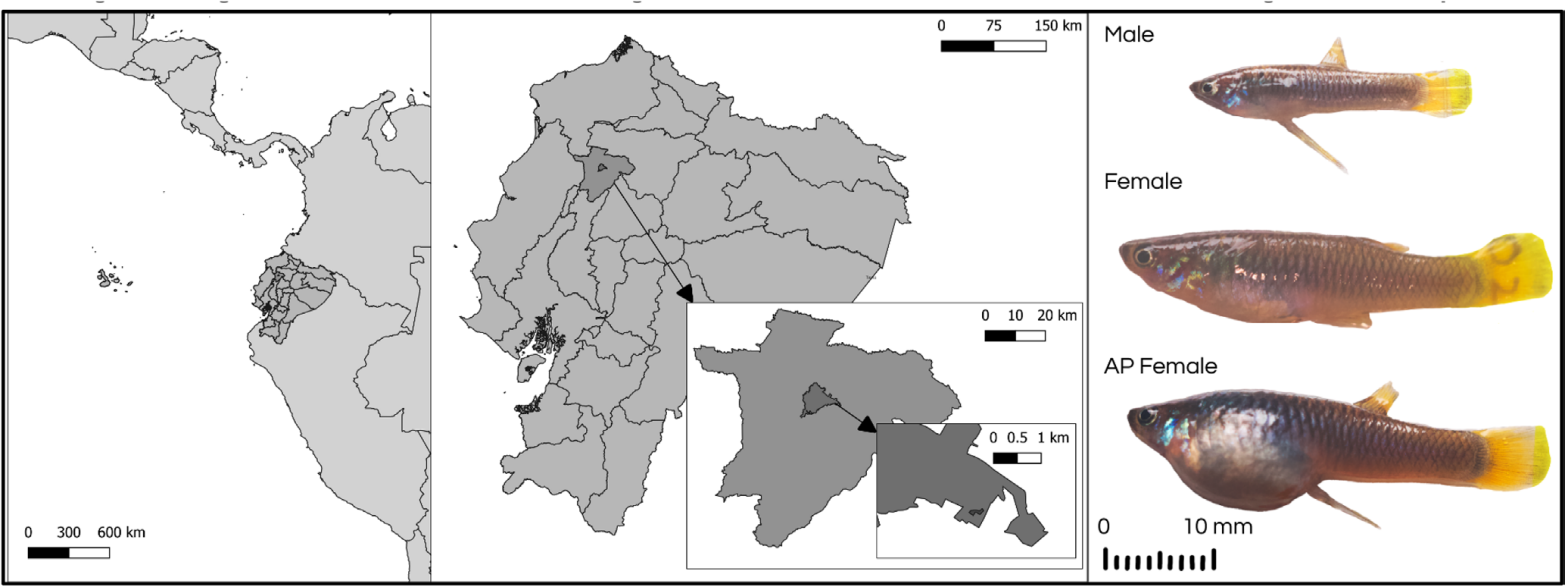
Left and center panels: Map showing the location of Santo Domingo de los Tsáchilas, the city in Ecuador where the population was encountered (indicated in the middle inserts). Right: Photographs of the three phenotypes encountered in the population, including male, female, and alternative phenotype female.

Unexpectedly, we discovered masculinized female *Pseudopoecilia fria* , a livebearing fish species in Santo Domingo de los Tsáchilas, Ecuador (Figure 1), while sampling for related projects. The fish were found in a low‐flowing stream located in a patch of secondary forest in an urban part of the Santo Domingo de los Tsáchilas Province. Upon visual inspection, we noticed females with gonopodia, modified anal fins by which male poeciliids transfer sperm (Gasparini et al. 2011). Gonopodia are male primary sexual characteristics found in all members of the family Poeciliidae with length and dimensions that vary dramatically among clades (Langerhans et al. 2005). *Pseudopoecilia fria* display varying degrees of sexual size dimorphism (SSD), but females are typically larger than males. Males are characterized by long gonopodia which, at times, reach approximately half their body length. Gonopodia are often extended while swimming, and, in addition to their obvious use as intromittent organs, could also function as highly salient visual signals (Langerhans 2011). Additionally, the hooks and barbs found at the end of gonopodia often function to grasp onto unreceptive females, allowing males to transfer sperm via coercive mating. Interestingly, one gonopodium‐bearing female was witnessed giving live birth to 8 babies, showing that they possess functional female organs. Here we assess phenotypic variation in *P. fria* by comparing different traits across sexual phenotypes (males, females, and masculinized females) and briefly explore possible consequences of this novel female phenotype.

## 2 Methods

### 2.1 Study Area

As part of a broader sampling effort, we identified a *P. fria* population within the city limits of Santo Domingo de los Tsáchilas, Ecuador (Figure 1). The focal population inhabits a roughly 200 m patch of secondary forest surrounded by an urban area, in a stream that is approximately 1 m wide with a depth of 20–60 cm. We visited the site during the dry season, so stream volume may increase with additional rainfall during the wet season. Anthropogenic waste was abundant on the higher edges of the creek, indicating that the stream could also have sewage effluent present.

### 2.2 Morphometric Measurements

The fish were anesthetized using 8–10 drops per 500 mL of a 1:10 dilution of clove oil (NOW) to facilitate photography, with images taken using a modified Canon EOS 7D camera with a metal bodied NIKKOR EL 80 mm lens and Baader UV‐IR blocking filter that allowed transmission from 420 to 680 nm. We took photographs of 37 individuals from Santo Domingo (17 males, 10 alternative phenotype females and 10 females). For the purposes of comparing morphology, we defined three a priori categories: *Males*, *Females* (females with standard fin phenotype) and *Alternative Phenotype females* (hereafter AP females: females with alterations to anal fins). To compare morphology between phenotypic classes, we performed morphometric measurements on a total of 30 individuals from Santo Domingo: 10 males, 10 females, and 10 alternative phenotype females. Five immature males were excluded from the morphometric analyses because the length of the gonopodium was not conclusively that of mature males, and one male with a thin gonopodium was excluded because it could be an uncommon variation in the population. Maturity was determined by examining gonopodia for the presence of hooks and the absence of an epithelial sheath using a Stereoscope ZEISS Stemi 305 at 1× magnification on anesthetized fish. We also excluded one mis‐identified male that later was confirmed to be an alternative phenotype female. To compare the morphology of the Santo Domingo population to a population without AP females, we additionally photographed 10 individuals (5 males and 5 females) from nearby dimorphic population, Nanegal. Initially, we measured standard length and gonopodium length. To quantify standard length, we used ImageJ 1.54p (Schneider et al. 2012) to measure the distance from the tip of the fish's snout to the beginning of the caudal fin. For gonopodium length we measured the length of the gonopodium from the ventral side of the fish's body to the tip of the gonopodium. To compare standard length and gonopodium length among phenotypic classes, we conducted Kruskal–Wallis tests and post hoc Dunn's tests in R version 4.5.0 (R Core Team 2025). Measurements were compared using a Kruskal‐Wallis test between sex phenotypes and populations (e.g., Santo Domingo males v. Nanegal males, Santo Domingo males v. Santo Domingo AP females). Additionally we took photos with a Microscope Olympus CX41 at 4× magnification of each fish's anal fin or gonopodium on both sides with the lateral side parallel with the microscope lens. To achieve clear images of the AP female gonopodium, we employed image stacking of different focal planes (from 4 to 6) (Figure 1).

In addition to taking basic morphometric measurements, we also asked whether AP female body shape also differs from that of other sexual phenotypes. We used ImageJ to establish 11 morphometric reference landmarks using methodology from Ardon et al. (2023) (illustrated in Figure 4A). We used MorphoJ v.1.08.02 (Klingenberg 2011) to assess geometric morphometrics to quantify phenotypic divergence between fish phenotypes using the a priori‐defined categories *Male*, *Female* and *AP female*. We employed generalized Procrustes analysis (GPA) to compare the average shapes of the different categories we specified based on landmark data. Then a Covariance Matrix was performed with Procrustes coordinates to perform a principal component analysis (PCA) to explore shape variation and identify which anatomical regions (i.e., landmarks) contributed most to the observed variance among individuals. To test which PC components explain most of the variation we used package *“paran”* (Dinno and Dinno 2018) in R to run a Horn's Parallel Analysis. Next, we run a Procrustes ANOVA to test if differences are significant between groups, and MorphoJ to calculate differences in the centroid size and shape. Then, we used the Procrustes aligned coordinates to test whether sexual phenotypes represent discrete phenotypic morphs with a canonical variate analysis (CVA) and identified the axes maximized shape variation between a priori groups. Finally, a Pairwise Procrustes Distance Matrix was constructed in R and we assessed the differences between morphs using 1000 permutations of a pairwise PERMANOVA with Bonferroni correction with the *“vegan”* package. Finally, homogeneity of multivariate dispersion was evaluated using *betadisper* to determine whether groups differed in within‐group shape variability, *PERMANOVA* could be sensitive to heterogeneity in dispersion. Group differences in dispersion were tested using an ANOVA.

## 3 Results

**FIGURE 2:**
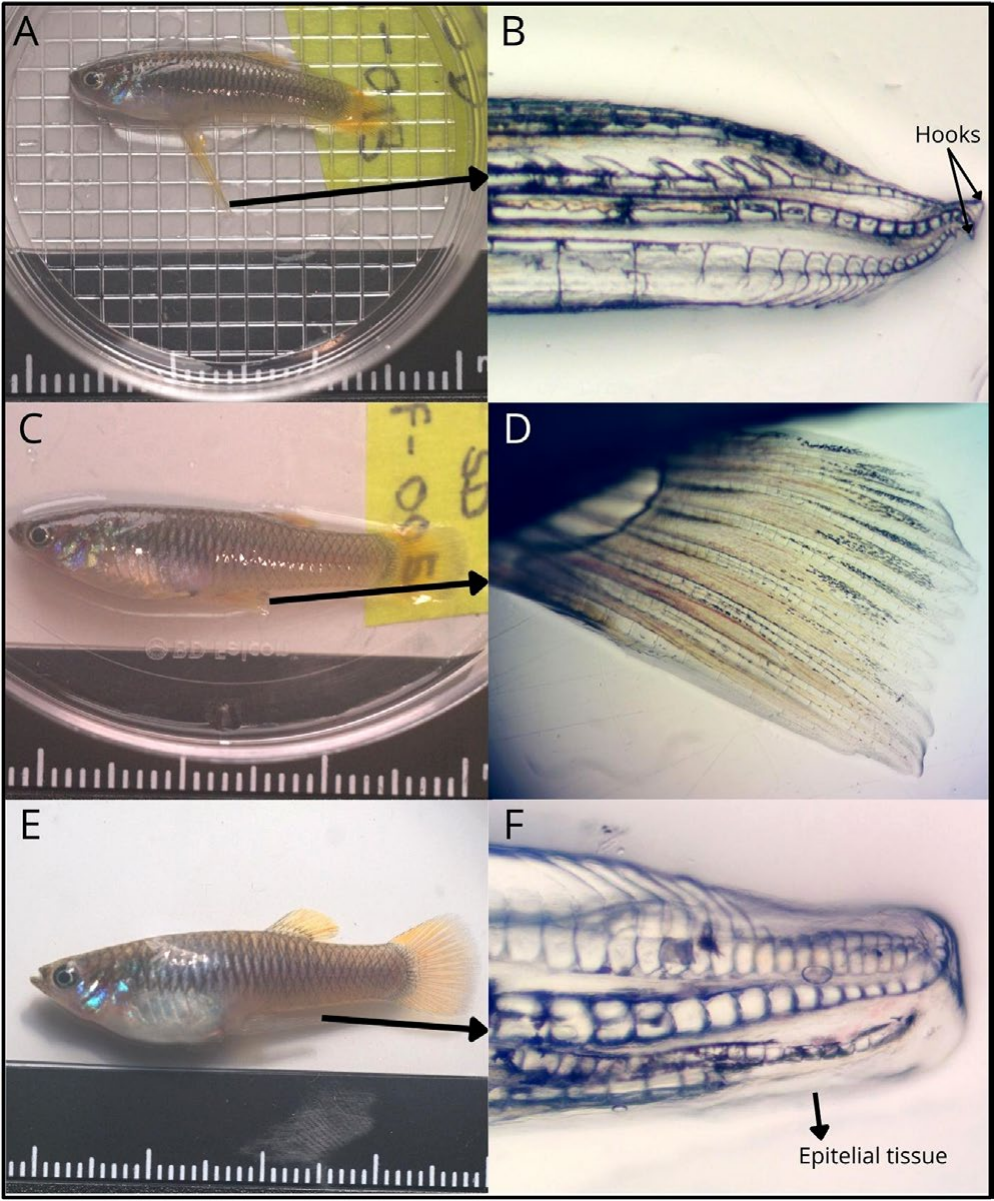
Full‐body and anal fin photographs of *Pseudopoecilia* phenotypes from Santo Domingo de los Tsáchilas. (A) Male phenotype with (B) normal (male) gonopodium exhibit two hooks (black arrows). (C) The standard female phenotype, (D) a typical female anal fin, and (E) the AP female phenotype with (F) modified gonopodium showing no hooks at the tip and an epithelial tissue that seems to cover the structures.

**FIGURE 3:**
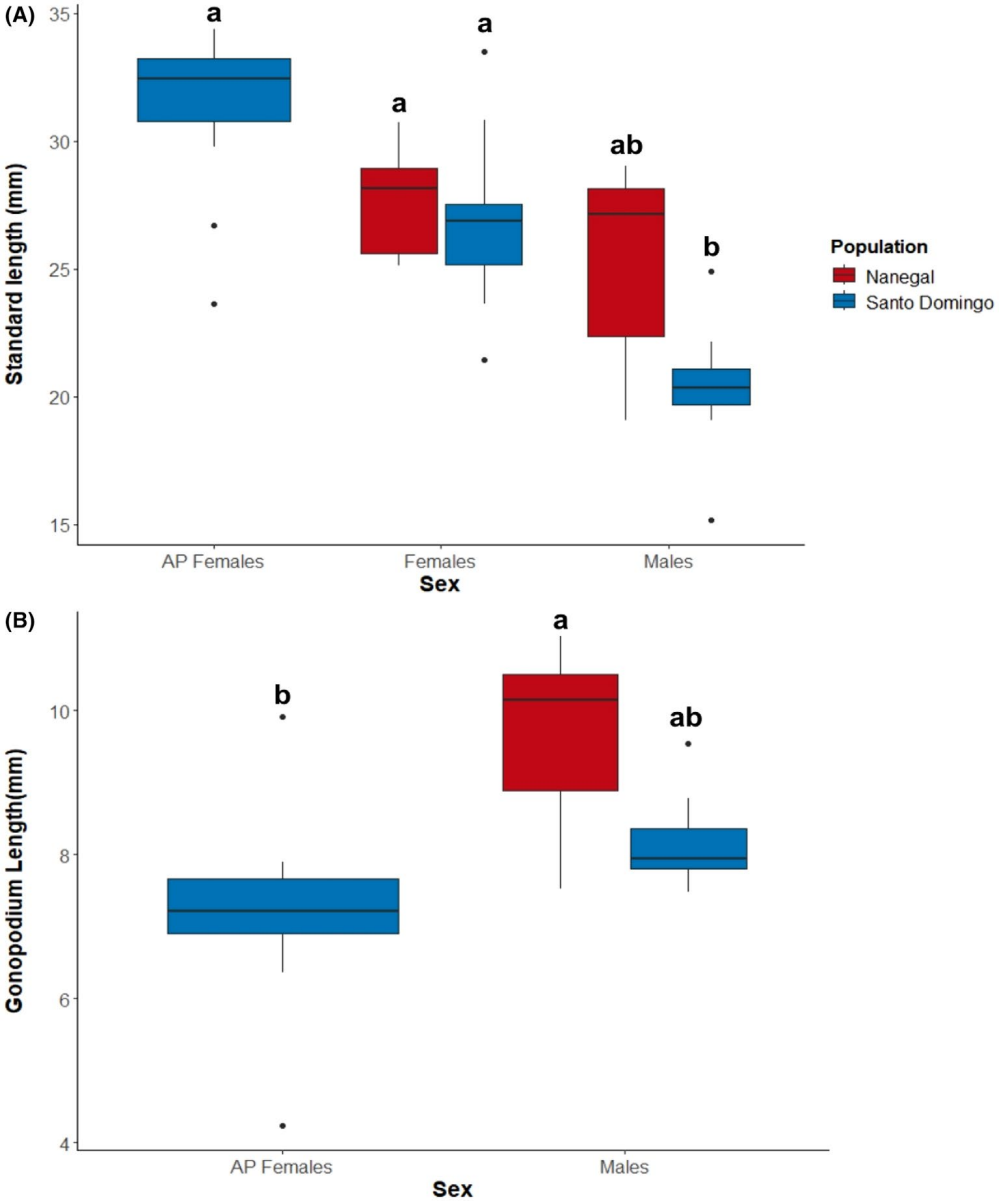
(A) Standard length (mm) comparison between Santo Domingo de los Tsáchilas (Blue) population and Nanegal (Red) population (121 km distance from Santo Domingo: Santo Domingo females [*n* = 10], Santo Domingo alternative phenotype (AP) females [*n* = 10], Santo Domingo males [*n* = 10], with Nanegal females [*n* = 5] and Nanegal males [*n* = 5]). (B) Comparison of gonopodium length (mm) between Santo Domingo males (*n* = 10), Nanegal males (*n* = 5), and AP females from Santo Domingo (*n* = 10).

We found that standard length differed among groups (Table 1) and between sexual phenotypes of the Santo Domingo de los Tsáchilas population (Figure 3A). AP females had higher standard length than Santo Domingo males, but not any of the other sexual phenotypes (Table 1). The comparison between mean gonopodium length of AP females and that of Santo Domingo males showed only a near‐significant trend in favor of males (*p* = 0.051). While Santo Domingo males did not differ from Nanegal males in gonopodium length, AP females showed significantly shorter gonopodia than Nanegal males (Figure 3B, Table 1). However, differences in gonopodium length between Nanegal males and AP females may also reflect population‐specific differences rather than differences between sexual phenotypes. In general, we can conclude that AP females tend to be similar in size to normally developing females, with a tendency towards shorter gonopodia than normally developing males. It is worth noting that AP female gonopodia are also not fully developed, lacking the two hooks found at the end of standard male gonopodia, but with additional epithelial tissue covering part of the gonopodium that is absent in adult males (Figure 2).

**TABLE 1:** Results of Kruskal–Wallis tests and post hoc Dunn tests (with Benjamini–Hochberg *p*‐value adjustment for multiple comparisons) comparing standard and gonopodium length across sexual phenotypes in the two populations. Codes for sexual phenotypes are AP Females (AP F), Females (F), and Males (M), while codes for populations are Santo Domingo (SD) and Nanegal (N).

Comparison	Kruskal–Walli's test across all sexual phenotypes	Post hoc Dunn's test between sexual phenotypes and populations				
		SD AP F—SD M	SD AP F—SD F	SD AP F—N M	AP F—N F	N M—SD M
Standard length across groups	H = 26.15	Z = 5.02	Z = 1.98	Z = 2.18	Z = 1.27	Z = 1.79
	***p* < 0.001**	***p* < 0.001**	*p* = 0.094	*p* = 0.073	*p* = 0.289	*p* = 0.123
Gonopodium length across groups	H = 28.02	Z = −2.23		Z = −2.44		Z = 1.33
	***p* < 0.001**	*p* = 0.051		***p* = 0.011**		*p* = 0.229

*Note:* Bold are significant values.

**FIGURE 4:**
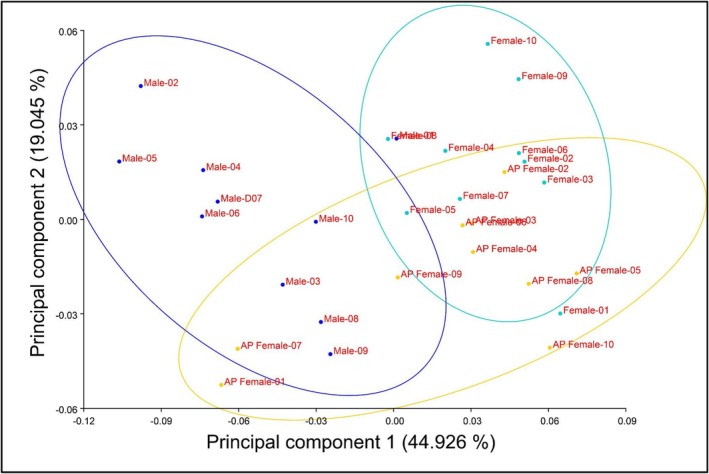
Principal component analysis (PCA) of 11 landmarks from 30 individuals from Santo Domingo to explore variation in body shape between individuals. Graphic shows confidence ellipses (90%) of Males (purple), Females (cyan) and AP females (yellow).

**FIGURE 5:**
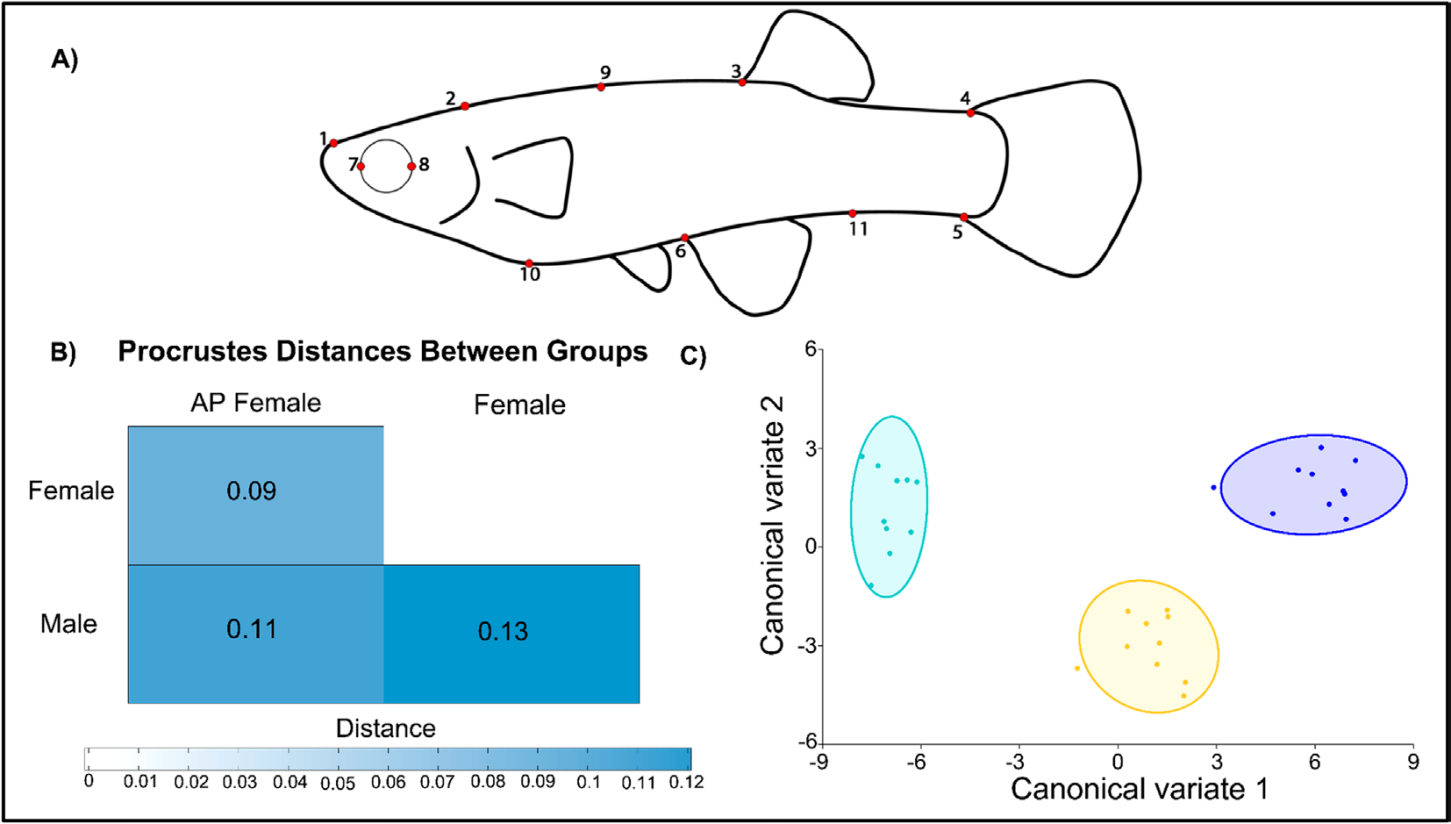
(A) Landmarks used for referencing anatomical regions in fish: (1) tip of the snout; (2) posterior extent of the operculum projected onto the dorsal outline; (3) anterior insertion of the dorsal fin; (4) dorsal insertion of the caudal fin; (5) ventral insertion of the caudal fin; (6) anterior insertion of the anal fin; (7) front of the eye; (8) back of the eye; (9) semilandmark on the dorsal outline halfway between landmarks 2 and 3; (10) semilandmark on the ventral outline at 2/3 the distance between landmarks 1 and 6; and (11) semilandmark on the ventral outline halfway between landmarks 5 and 6 (following Ardon et al. 2023). (B) Pairwise distances matrix show distances between each phenotype. Canonical Variate Analysis (CVA) of 11 landmarks from the 30 individuals from Santo Domingo individuals (*n* = 10 each of AP females, normal females, and males). Graphic shows 90% confidence ellipses for Males (Blue), Females (turquoise) and AP females (yellow).

### 3.1 Morphometric Analyses

The Horn's Parallel Analysis confirmed that only PC1 and PC2 had eigenvalues > 1 (PC1 = 4.5066, PC2 = 1.7548), the cumulative morphological variance explained by these components was 63.98% (44.93% and 19.05% respectively; Figure 4). Males and females grouped assortatively into negative and positive values for PC1 respectively, where AP variance was higher and intermediate overlapped with both positive and negative values. Procrustes ANOVA results indicated significant morphological differences between the groups (Table S1; Centroid size: *p* < 0.001, *F =* 2.83; Shape: *p* < 0.001, *F* = 13.604, Pillai's traces = 1.81) and CVA shows high variation in morphology between groups (Figure 5C). Together this suggests strong differentiation in morphology associated with distinct sexual phenotypes reinforcing the hypothesis that sexual phenotypes indeed represent discrete morphs.

The pairwise distances matrix (Figure 5B) shows that AP females are more similar to females than to males in the population in terms of overall body shape, and the three morphs significantly differ from one another. Pairwise *PERMANOVA* shows distances among the sexual phenotypes are significant (Table S2; AP‐F vs. F: *p‐adjusted* = 0.003, AP‐F vs. M: *p‐adjusted* = 0.006, F vs. M: *p‐adjusted* = 0.003). No significant differences were detected in multivariate dispersion between groups (ANOVA, *p* = 0.29), indicating homogeneity in intragroup variability. Our results suggest that the categories we selected for the analysis of this case could represent morphological discrete phenotypes in the Santo Domingo population.

## 4 Discussion

Cross‐sexual transfer of a male reproductive organ to females in a population of live‐bearing fish is unusual and offers unique opportunities to study the causes and consequences of this variation. We tentatively assign female masculinization as the most likely mechanism to explain this phenomenon, yet it may have occurred through a number of different pathways. One potential explanation involves differential sensitivity to environmental contamination among females, which would explain the presence of AP females among standard females. The population was discovered in an urban area with a high density of industrial factories, as well as mining. If alternate female phenotypes are due to environmental causes, then the presence of this alternate morph may be transitory and could be lost if the point source of contamination disappeared (Parks et al. 2001; Larsson et al. 2002). While the two phenotypic classes are syntopic, females might still exhibit dosage‐dependent responses due to different levels of exposure based on state‐behavior feedback loops (Sih et al. 2015) or caused by episodic pollution during sensitive developmental periods (Seager and Maltby 1989; Handy 1994). We also cannot rule out a potential contribution of dispersal between contaminated and uncontaminated environments to explain differences in the presence and absence of masculinization among Santo Domingo females.

In the related species *Gambusia holbrooki* , prolonged exposure to synthetic hormones produced morphological changes to anal fins which resulted in gonopodium‐like protrusions in as short as 9–11 days post‐exposure (Tran et al. 2022), which supports the hypothesis that masculinization could be potentially attributable to water‐based pollution, and could occur rapidly as a plastic response to environmental conditions. Notably, similar to fish from the Tran et al. (2022) study, the alternative morph females we encountered did not have fully developed gonopodia, lacking the hook on the tip of the structure (Figure 4). Because hooks may be used by males to transfer sperm to unreceptive females in other poeciliids (Langerhans 2011; Kwan et al. 2013), the absence of normally developed hooks indicates that the gonopodia may not be reproductively functional. This reinforces our tentative hypothesis that a cross‐sexual transfer in the form of masculinization, where remediation in the local environment would likely attenuate the frequency of gonopodia in females. However, the timescale of this change will require further study.

As an alternative to an environmental cause, it is also plausible that the existence of two female morphs could be attributable to a genetic mechanism including but not limited to mutations (Jeong et al. 2022), or polyploidy (Lampert et al. 2008). If this were the case, then the presence of bimorphic females could potentially represent a stable polymorphism or even an alternative reproductive tactic akin to those found in many male poeciliid fish (Farr et al. 1986; Ryan and Causey 1989; Erbelding‐Denk et al. 1994; Hurtado‐Gonzales and Uy 2009; Liotta et al. 2019).

Females with male sexual characteristics present an interesting opportunity to study both inter‐ and intrasexual social interactions. A remote possibility is that a subset of females have evolved intersexual mimicry of males by developing nonfunctional gonopodia. Among other poeciliids, sexual mimicry has so far only been documented in some *Xiphophorus* species where males develop false gravid spots typical of mated females which reduce male–male aggression, but higher rates of sexual attention from other males in species with high levels of male–male agonism (Rios‐Cardenas et al. 2010; Dodge et al. 2024). Alternative morph females sampled showed visible evidence of pregnancy, which implies that they are not always mistaken for males, in spite of their salient gonopodia. Perhaps this is because, like coercive males in other poeciliids (Farr and Travis 1986; Furness et al. 2020), *P. fria* males are indiscriminate in mating, though this warrants further research.

Notably, our observation of this female phenotype is limited to a single population, and we lack knowledge of behavioral interactions as well as proximate mechanisms underlying the morph. Therefore, any adaptive or mechanistic hypothesis is highly speculative. Future study would be important to determine if any adaptive benefits exist, or if female masculinization only incurs fitness costs. Measuring male interactions in social settings including both female morphs could afford insights into whether males adjust their mating behavior to female morphs, as well as the receptivity of both female morphs to coercive male mating attempts. Alterations in the mating behavior of masculinized females and male receptivity to them may be another interesting and unexpected consequence of anthropogenic change on reproductive traits (e.g., Ramirez‐Duarte et al. 2025).

Alternative morph females with male gonopodia are an unexpected and unique finding in *Pseudopoecilia* and among poeciliid fish generally. Future work is necessary to identify the cause(s) of this polymorphism (environmental, genetic, or both) which will clarify the mechanisms underlying the development of alternative morph females. Just as field studies have shed light on the unusual unisexual‐bisexual systems found in other poeciliids (Moore 1975; Hubbs and Schlupp 2008; Schlupp 2010), long‐term monitoring of the population could also afford important insights into the dynamics or stability of this alternative female phenotype. Similarly, tracking AP female offspring to sexual maturity will be useful in determining the heritability of this phenotype. So far, our findings add to a growing literature on cross‐sexual transfer that highlights the astonishing developmental plasticity and evolutionary lability of sexual traits (Anderson and Falk 2023; Robinson et al. 2023; Friberg et al. 2025). Behavioral studies will clarify the intra‐ and intersexual dynamics between males, females and alternative phenotype females which will be important in understanding the evolutionary consequences to their persistence.

## Author Contributions

**Justin Yeager:** conceptualization (lead), formal analysis (equal), funding acquisition (equal), investigation (equal), methodology (equal), project administration (lead), resources (lead), supervision (lead), validation (equal), writing – original draft (lead), writing – review and editing (lead). **Leonardo Avila:** data curation (equal), formal analysis (equal), methodology (equal), writing – original draft (supporting), writing – review and editing (equal). **Callen Inman:** formal analysis (equal), investigation (equal), validation (equal), writing – review and editing (equal). **Marissa Cartee:** data curation (equal), formal analysis (equal), methodology (equal), supervision (equal), writing – review and editing (equal). **Micaela Pozo:** formal analysis (equal), investigation (equal), writing – review and editing (supporting). **Dillan Burbano:** investigation (equal), methodology (equal), writing – review and editing (supporting).

## Funding

This work was supported by UDLA Grant, UDLA grant 483.A.XIV.24.

## Conflicts of Interest

The authors declare no conflicts of interest.

## Supporting information


**Table S1:** Results of procrustes ANOVA test on PC centroid size and shape across groups.
**Table S2:** Pairwise PERMANOVA test on procrustes distance matrix assessing differences among sexual phenotypes in the Santo Domingo population.

## Data Availability

Data are publicly available: https://github.com/inmanc2/pseudo_females_with_gonopodia.
