# Supplementary material for: Male Copulatory Structures in Reproductively Functional Female Live‐Bearing Fish Pseudopoecilia fria
Source: Ecol Evol. 2026 Feb 17;16(2):e73118. doi: 10.1002/ece3.73118 (PMC12912842; doi:10.1002/ece3.73118)
Supplement: Supplementary file 1 — Table S1: Results of procrustes ANOVA test on PC centroid size and shape across groups. Table S2: Pairwise PERMANOVA test on procrustes distance matrix assessing differences among sexual phenotypes in the Santo Domingo population. [file ECE3-16-e73118-s001.docx]

**Supplementary Table 1**. Results of Procrustes ANOVA test on PC centroid size and shape across groups.

| **Procrustes ANOVA** | | | | | | |
| --- | --- | --- | --- | --- | --- | --- |
|  | ***SS*** | ***MS*** | ***df*** | ***F*** | ***P*** | ***Pillai’s trace*** |
| Centroid size | 824.4064 | 412.3032 | 2 | 28.83 | <0.001 |  |
| Shape | 0.0806 | 0.0022 | 36 | 13.60 | <0.001 | 1.81 |
| **Supplementary Table 2.** Pairwise PERMANOVA test on Procrustes Distance Matrix assessing differences among sexual phenotypes in the Santo Domingo population.  **Pairwise PERMANOVA** | | | | | | |
| ***Pair*** | | | ***F Model*** | ***R^2^*** | ***p-value*** | ***p-adjusted*** |
| AP Female vs Female | | | 7.5021 | 0.2942 | 0.00099 | 0.00299 |
| AP Female vs Male | | | 10.5218 | 0.3689 | 0.00198 | 0.00599 |
| Female vs Male | | | 24.6853 | 0.5783 | 0.00099 | 0.00299 |
|  |  |  |  |  |  |  |
|  |  |  |  |  |  |  |
